# Supplementary material for: Licorice Extract Supplementation Benefits Growth Performance, Blood Biochemistry and Hormones, Immune Antioxidant Status, Hindgut Fecal Microbial Community, and Metabolism in Beef Cattle
Source: Vet Sci. 2024 Aug 6;11(8):356. doi: 10.3390/vetsci11080356 (PMC11359752; doi:10.3390/vetsci11080356)
Supplement: Supplementary file 1 [file vetsci-11-00356-s001.zip › vetsci-3115258-supplementary.pdf]

Table S1. Basis of fodder and nutrient levels (%)

Item	Time		
	0-30 d	30-60 d	60-90 d
**Ingredients**			
Silage	25.37	45.63	42.06
Rice straw	37.31	24.27	19.84
Fragrant thatch	0.00	0.00	3.97
Cotton seed	7.46	5.83	9.52
Corn	10.45	8.50	8.61
Soybean meal	10.45	8.50	8.61
Wheat bran	7.16	5.83	5.90
Sodium bicarbonate	0.60	0.49	0.49
Premix	1.19	0.97	0.98
Total	100	100	100
**Nutrient level**			
CP	14.81	15.08	13.89
NDF	50.99	46.42	50.90
ADF	35.73	28.92	34.70
Ca	1.24	1.43	1.31
P	0.18	0.18	0.20
EE	15.38	14.51	15.70

Per kg premix contains: VA 100-600 KIU, VD3 30-200 KIU, VE 220 mg, Cu 100-500 mg, Fe 1000-15000 mg, Mn 200-3000 mg, Zn 1000-2200 mg, Ca 5%-15%, P 1.5%-5%, NaCl 5%-25%.

Table S2. The influence of licorice extract on OUTs and alpha diversity parameters of beef cattle hindgut fecal bacterial community sequencing.

Item	Group	
	CK	CHM
OTUs	1224.00±41.60	1215.83±56.16
ACE	1240.55±42.17	1227.05±56.62
Chao 1	1237.39±41.55	1222.73±56.62
Shannon	8.87±0.12	9.00±0.82
Simpson	0.99±0.00	1.00±0.00

CK=control group; CHM=Licorice extract group.

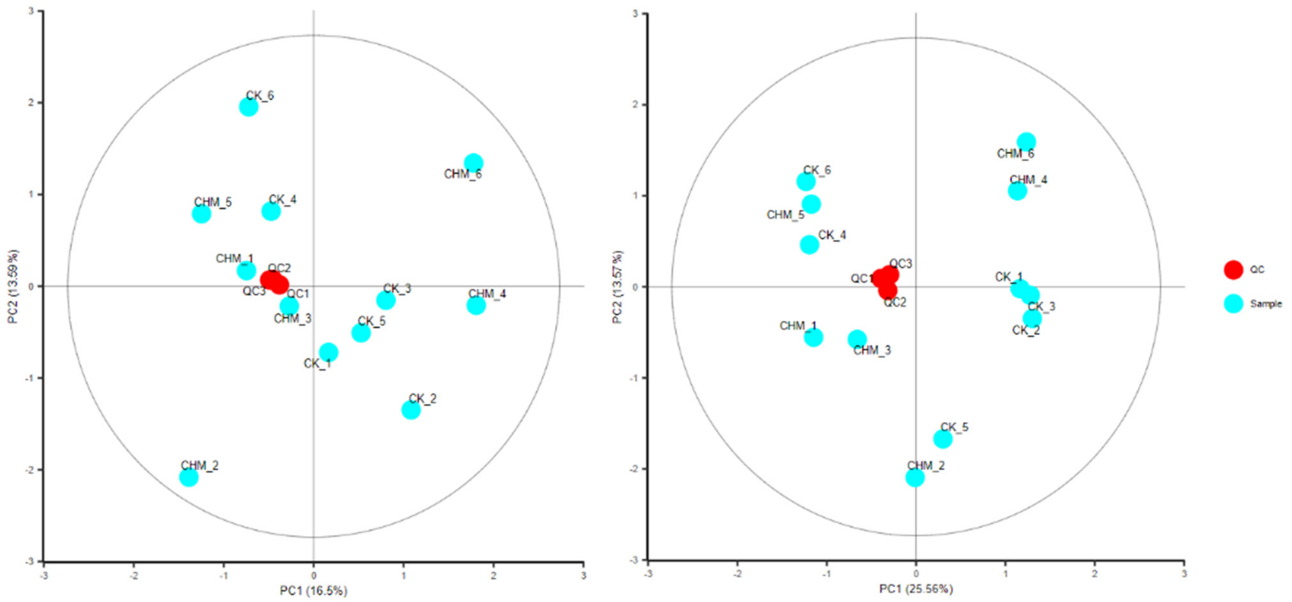

(a) (b)

Figure S1. Principal component analysis (PCA) plot of data quality control for the fecal metabolome of beef cattle. (**a**) Quality control chart for positive ions. (**b**) Quality control chart of negative ion mode.The samples in the cation and anion modes were relatively close, showing good reproducibility and stability of the LC-MS method.CK and CHM respectively represent the control group and the experimental group.
